# Impact of infections on the incidence of acute inflammatory demyelinating polyneuropathy in children

**DOI:** 10.1002/cns3.20022

**Published:** 2023-05-03

**Authors:** Hannah Gilbert, Nicholas S. Abend, Melissa Hutchinson, Ricka Messer, Mahendranath Moharir, Kendall Nash, Jamie Palaganas, Juan Piantino, Samir S. Shah, Matt Hall, Elizabeth Wells, Craig A. Press, Pediatric Neurohospitalist Work Group

**Affiliations:** ^1^ Department of Pediatrics, Section of Child Neurology Children's Hospital Colorado and the University of Colorado Aurora Colorado USA; ^2^ Departments of Neurology and Pediatrics Children's Hospital of Philadelphia and the University of Pennsylvania Philadelphia Pennsylvania USA; ^3^ Department of Pediatrics, Neurology Division The Ohio State University College of Medicine, Nationwide Children's Hospital Columbus Ohio USA; ^4^ Department of Pediatrics, Division of Neurology The Hospital for Sick Children and University of Toronto Toronto Ontario Canada; ^5^ Departments of Neurology and Pediatrics, Division of Child Neurology, Benioff Children's Hospital San Francisco University of California, San Francisco San Francisco California USA; ^6^ Department of Pediatrics, Division of Child Neurology Weill Cornell Medicine, New York Presbyterian Hospital New York New York USA; ^7^ Divisions of Hospital Medicine and Infectious Diseases Cincinnati Children's Hospital Medical Center and the University of Cincinnati College of Medicine Cincinnati Ohio USA; ^8^ Department of Pediatrics, Section of Child Neurology Oregon Health & Science University Portland Oregon USA; ^9^ Children's Hospital Association Lenexa Kansas USA; ^10^ Center for Neuroscience and Behavioral Medicine, Children's National Hospital and the George Washington University School of Medicine and Health Sciences Washington District of Columbia USA

**Keywords:** acute inflammatory demyelinating polyneuropathy, COVID‐19, Guillain–Barre syndrome, infection, pediatric

## Abstract

**Objectives:**

Acute inflammatory demyelinating polyneuropathy (AIDP) is the leading cause of acute flaccid paralysis in children and hypothesized to be triggered by antecedent infection. We sought to determine the association between AIDP and commonly acquired community infections in children. We utilized the reduction in these infections due to measures during coronavirus disease 2019 (COVID‐19) to serve as a natural experiment and determine their contribution to AIDP.

**Methods:**

This cross‐sectional study used administrative and billing data from children's hospitals contributing to the Pediatric Health Information System. We included hospitalizations of children with a diagnosis of AIDP from (January 2017 through February 2021). Encounters for infection‐ (including respiratory, gastrointestinal, and COVID‐19) related diagnoses were measured as a marker of community incidence.

**Results:**

A total of 1111 index encounters for AIDP were included. Pre‐COVID‐19, AIDP was not associated with respiratory or gastrointestinal infections, specifically, influenza or campylobacter. During the COVID‐19 period from March 2020 to February 2021, respiratory, gastrointestinal, and influenza infections decreased compared to expected (for the same time of year pre‐COVID‐19) by 59.6%–90.1%, 51.5%–68.9%, and 54.5%–97.9%, respectively. In contrast, AIDP hospitalizations and all hospitalizations only decreased by 11.5%–39.3% and 14.2%–25%, respectively. COVID‐19 was not positively associated with AIDP overall or at individual hospitals.

**Interpretation:**

Common community‐acquired infections including COVID‐19 were not strongly associated with hospitalizations for AIDP in children. AIDP persisted despite the dramatic reduction in infection‐related encounters during the pandemic. These results suggest that recent antecedent community‐acquired infections were not the primary driver of AIDP and that alternative triggers should be explored.

## Introduction

Acute inflammatory demyelinating polyneuropathy (AIDP), the predominant form of Guillain–Barre syndrome in the Western world, is the leading cause of acute flaccid paralysis in children.^1^ The pathogenesis of AIDP is hypothesized to be driven by an antecedent infection that triggers an immune response against cross‐reactive epitopes.^2^ The strongest evidence is related to infection with *Campylobacter jejuni* (*C. jejuni*).^3^ Additional infections, including influenza, are cited both in the media and scientific publications despite limited evidence.^4^ Case reports in adults and children have suggested a link between coronavirus disease 2019 (COVID‐19) caused by the severe acute respiratory syndrome coronavirus‐2 (SARS CoV‐2) infection and AIDP.^5^, ^6^, ^7^

We sought to determine the association between the incidence of infections and the incidence of AIDP. We took advantage of the natural experiment facilitated by the preventive measures put in place during the COVID‐19 pandemic causing widespread decreases in community‐acquired infections to explore the link between community‐acquired infections and hospitalizations for AIDP in children.

## Methods

### Data source

This cross‐sectional study used data from Pediatric Health Information System (PHIS), an administrative database containing inpatient, observation, emergency room (ER), and ambulatory surgery data from 47 United States tertiary children's hospitals (Children's Hospital Association, Lenexa, KS). For each encounter, PHIS includes demographic information and a maximum of 41 ICD‐10 (International Statistical Classification of Diseases and Related Health Problems) diagnosis codes. Daily billing data is collected for each encounter and patients can be tracked longitudinally across encounters using a unique encrypted patient identifier.

### Main exposures

The main exposure was the incidence of infection encounters (ER, inpatient, and observation), including respiratory infections, gastrointestinal infections, other viral infections (cytomegalovirus [CMV] and Epstein–Barr virus [EBV]), and COVID‐19/multisystem inflammatory syndrome in children (MIS‐C) (Supporting Information: Table 1). All encounter types were included to identify all encounters for infections as a marker of community spread. Data extracted included demographics, level of care (intensive care unit [ICU] vs. non‐ICU), days ventilated, and treatments for AIDP, including intravenous immune globulin (IVIG) and/or plasmapheresis/plasma exchange (PLEX).

### Main outcomes

The main outcome was the volume of index AIDP encounters. We included all hospitalizations (inpatient and observation) of children >30 days old and <26 years old with a discharge diagnosis of AIDP using the ICD‐10 code G610, from January 1, 2017 to February 28, 2021. Only hospitals that participated in PHIS throughout this timeframe were included. Encounters were excluded for patients with alternative AIDP secondary diagnoses: encephalitis, myelitis and encephalomyelitis (G04), transverse myelitis (G37), optic neuritis (G36, H46), multiple sclerosis (MS) (G35), botulism (A48.51, A48.52, A05.1), and tic paralysis (toxic effect of venom of other arthropod) (T63.48). We selected additional encounter diagnoses to serve as controls for conditions that would be likely or unlikely to be impacted by a reduction in community‐acquired infections and would also still likely present to care. The “positive control” for infection‐related AIDP was asthma, defined by the presence of discharge diagnosis codes for asthma acute exacerbations (J45.*1, J45.901) or status asthmaticus (J45.*2, J45.902) (positive control for association with infections). The “negative controls” unlikely to be infection related were index encounters for brain tumors (C71, C72) or diabetic ketoacidosis (E101). We categorized associations as strong (*r* > 0.7), moderate (*r* = 0.5–0.7), weak (*r* = 0.3–0.49), or very weak (*r* < 0.3).

### Time periods

We defined the pre‐COVID‐19 time period as inclusive of January 2017 through February 2020. The COVID‐19 time period included March 2020 through February 2021, which was broken down into four‐month blocks accounting for potential effects of variation in social distancing, the incidence of COVID‐19, and historical infectious trends (e.g., influenza season).

### Statistical analyses

Categorical variables were summarized using frequencies with percentages, and continuous variables were summarized using medians and interquartile ranges. Comparisons across periods were made with *χ* ^2^ tests. Median monthly volumes in COVID‐19 months were compared to prior years using median regression. Correlations between volumes were assessed using Spearman's correlation. Correlations were assessed in aggregate and by the hospital for COVID‐19/MIS‐C. All analyses were performed using SAS version 9.4 (SAS Institute Inc.). *p* values < 0.05 were considered statistically significant.

### Standard protocol approvals, registrations, and patient consents

The study was approved by the University of Colorado Institutional Review Board with a waiver of informed consent.

## Results

### AIDP hospitalization demographics and treatment

A total of 1111 index encounters for AIDP were included in this study (905 pre‐COVID‐19 and 206 during the COVID‐19 pandemic) after excluding 65 encounters (Table 1). There was a significant difference in age over time with a trend toward older patients during the pandemic period. There were no significant differences in sex, race, payor, total length of stay, ICU length of stay, or days of mechanical ventilation between time periods. AIDP during index hospitalizations pre‐COVID‐19 was predominantly treated with IVIG (60.3%), and overall, there was no change in treatment during the pandemic (*p* = 0.051). There was an increased proportion of hospitalizations where neither IVIG nor PLEX was utilized from November 2020 to February 2021 compared to the same period in prior years (+46.7%, *p* < 0.001; Table 2).

**Table 1:** Demographics of AIDP encounters by time period.

		Pre‐COVID‐19	COVID‐19 pandemic			
	Overall	Jan 2017–Feb 2020	Mar–Jun 2020	Jul–Oct 2020	Nov 2020–Feb 2021	*p* value
*N*	1111	905	69	73	64	
Age	10 [4, 14]	9 [4, 14]	12 [5, 17]	12 [8, 15]	11 [8, 16]	**<0.001**
Age category (years)						**<0.001**
0	12 (1.1)	12 (1.3)	0 (0.0)	0 (0.0)	0 (0.0)	
1–4	277 (24.9)	246 (27.2)	17 (24.6)	8 (11)	6 (9.4)	
5–9	251 (22.6)	209 (23.1)	10 (14.5)	18 (24.7)	14 (21.9)	
10–14	309 (27.8)	242 (26.7)	14 (20.3)	26 (35.6)	27 (42.2)	
15–21	262 (23.6)	196 (21.7)	28 (40.6)	21 (28.8)	17 (26.6)	
Gender						0.189
Male	608 (54.7)	500 (55.2)	35 (50.7)	33 (45.2)	40 (62.5)	
Female	503 (45.3)	405 (44.8)	34 (49.3)	40 (54.8)	24 (37.5)	
Race						0.151
Non‐Hispanic White	558 (50.2)	444 (49.1)	45 (65.2)	35 (47.9)	34 (53.1)	
Non‐Hispanic Black	146 (13.1)	117 (12.9)	11 (15.9)	12 (16.4)	6 (9.4)	
Hispanic	295 (26.6)	246 (27.2)	10 (14.5)	19 (26)	20 (31.3)	
Asian	27 (2.4)	25 (2.8)	0 (0.0)	0 (0.0)	2 (3.1)	
Other	85 (7.7)	73 (8.1)	3 (4.3)	7 (9.6)	2 (3.1)	
Payor						0.130
Government	565 (50.9)	448 (49.5)	32 (46.4)	43 (58.9)	42 (65.6)	
Private	486 (43.7)	407 (45)	34 (49.3)	27 (37)	18 (28.1)	
Other	60 (5.4)	50 (5.5)	3 (4.3)	3 (4.1)	4 (6.3)	
Encounter variables						
LOS (days)	5 [3, 13]	6 [3, 13]	5 [3, 12]	5 [3, 16]	4 [2, 9]	0.148
Required ICU care	267 (24)	219 (24.2)	20 (29)	17 (23.3)	11 (17.2)	0.457
ICU LOS (days)	4 [2, 9]	4 [2, 8]	8 [3, 14]	6 [3, 9]	3 [1, 9]	0.151
Required mech vent	122 (11)	96 (10.6)	11 (15.9)	8 (11)	7 (10.9)	0.601
Days of vent	7 [3, 14]	7 [3.5, 16]	7 [3, 11]	3.5 [1, 15]	3 [2, 9]	0.275
Treatments						0.051
Only IVIG	670 (60.3)	550 (60.8)	46 (66.7)	46 (63)	28 (43.8)	
Only PLEX	34 (3.1)	32 (3.5)	0 (0.0)	1 (1.4)	1 (1.6)	
IVIG and PLEX	56 (5)	43 (4.8)	4 (5.8)	6 (8.2)	3 (4.7)	
Neither	351 (31.6)	280 (30.9)	19 (27.5)	20 (27.4)	32 (50)	

Bold values are statistically significant <0.05.

AIDP, acute inflammatory demyelinating polyneuropathy; COVID‐19, coronavirus disease 2019; ICU, intensive care unit; IVIG, intravenous immunoglobulin; LOS, length of stay; mech, mechanical; PLEX, plasma exchange/plasmapheresis; vent, ventilation.

**Table 2:** Percentage change in the median monthly volume of encounters by time period related to COVID‐19.

	Mar–Jun 2020		Jul–Oct 2020		Nov 2020–Feb 2021	
	Percentage change in medians (95% CI)	*p* value	Percentage change in medians (95% CI)	*p* value	Percentage change in medians (95% CI)	*p* value
All ER/obs/admissions	**−46.1 (−85.4, −6.8)**	**0.025**	**−28.9** (**−38.9, −19)**	**<0.001**	**−43.5 (−49.4, −37.5)**	**<0.001**
All admissions	−21.8 (−50.1, 6.6)	0.122	**−14.2 (−18.4, −9.9)**	**<0.001**	**−25 (−29.9, −20)**	**<0.001**
Encounters for						
All respiratory	−74.8 (−238, 88.4)	0.342	**−59.6 (−105.4, −13.7)**	**0.015**	**−90.1 (−115.2, −65)**	**<0.001**
Influenza	−54.5 (−686.1, 577)	0.856	−79.6 (−177.7, 18.5)	0.104	**−97.9 (−181.6, −14.3)**	**0.025**
RSV	−56.8 (−423.8, 310.2)	0.745	−72.9 (−210.1, 64.4)	0.274	**−98.4 (−123.2, −73.7)**	**<0.001**
Mycoplasma	−29.1 (−223.3, 165.1)	0.753	**−67.7 (−118.7, −16.6)**	**0.013**	**−76.6 (−114.9, −38.4)**	**0.001**
All gastrointestinal	**−68.9 (−125.5, −12.2)**	**0.021**	**−51.5 (−62, −41.1)**	**<0.001**	**−63.6 (−81.2, −46)**	**<0.001**
*Campylobacter*	−21.3 (−116.2, 73.6)	0.637	10.3 (−47.8, 68.3)	0.710	−9.7 (−76.9, 57.6)	0.764
Rotavirus	**−87.2 (−154.4, −20)**	**0.015**	−33.3 (−89.6, 23)	0.225	−69.8 (−195, 55.5)	0.255
CMV	−10.3 (−42.3, 21.7)	0.500	−4.8 (−28, 18.4)	0.663	−15.4 (−39, 8.2)	0.186
EBV	**−21.3 (−38.9, −3.7)**	**0.021**	−3.3 (−24.6, 18.1)	0.748	−3.3 (−20.2, 13.7)	0.689
AIDP						
All index encounters	−30.5 (−68.9, 7.9)	0.110	−11.5 (−44.7, 21.6)	0.468	−39.3 (−86, 7.4)	0.094
Requiring ICU	−46.2 (−106.1, 13.8)	0.121	0 (−119.8, 119.8)	1.000	−25 (−77.1, 27.1)	0.324
Requiring ventilation	−40 (−188.8, 108.8)	0.574	−33.3 (−125.1, 58.5)	0.447	−50 (−279, 179)	0.647
Treatment IVIG and/or PLEX	−17.1 (−54.8, 20.5)	0.345	−18.8 (−39.9, 2.4)	0.078	**−46.7 (−68.9, −24.4)**	**<0.001**
Non‐AIDP encounters						
Index encounters brain tumor	**−33.4 (−53.1, −13.7)**	**0.003**	−9.4 (−39.2, 20.4)	0.509	−20 (−52.2, 12.2)	0.207
Index encounters for DKA	−14.9 (−41.1, 11.4)	0.245	2 (−13.5, 17.5)	0.787	−2 (−16.4, 12.5)	0.778
Asthma	**−83.3 (−148, −18.6)**	**0.015**	**−57.5 (−104.2, −10.8)**	**0.019**	**−69 (−87.8, −50.3)**	**<0.001**

Bold values are statistically significant <0.05.

AIDP, acute inflammatory demyelinating polyneuropathy; *Campylobacter*, *campylobacter jejunii*; CI, confidence interval; CMV, cytomegalovirus; COVID‐19, coronavirus disease 2019; DKA, diabetic ketoacidosis; EBV, Epstein–Barr virus; ER, emergency room; ICU, intensive care unit; IVIG, intravenous immunoglobulin; Obs, observation; PLEX, plasma exchange/plasmapheresis; RSV, respiratory syncytial virus.

### AIDP hospitalization and association with infectious encounters pre‐COVID‐19

**Figure 1:**
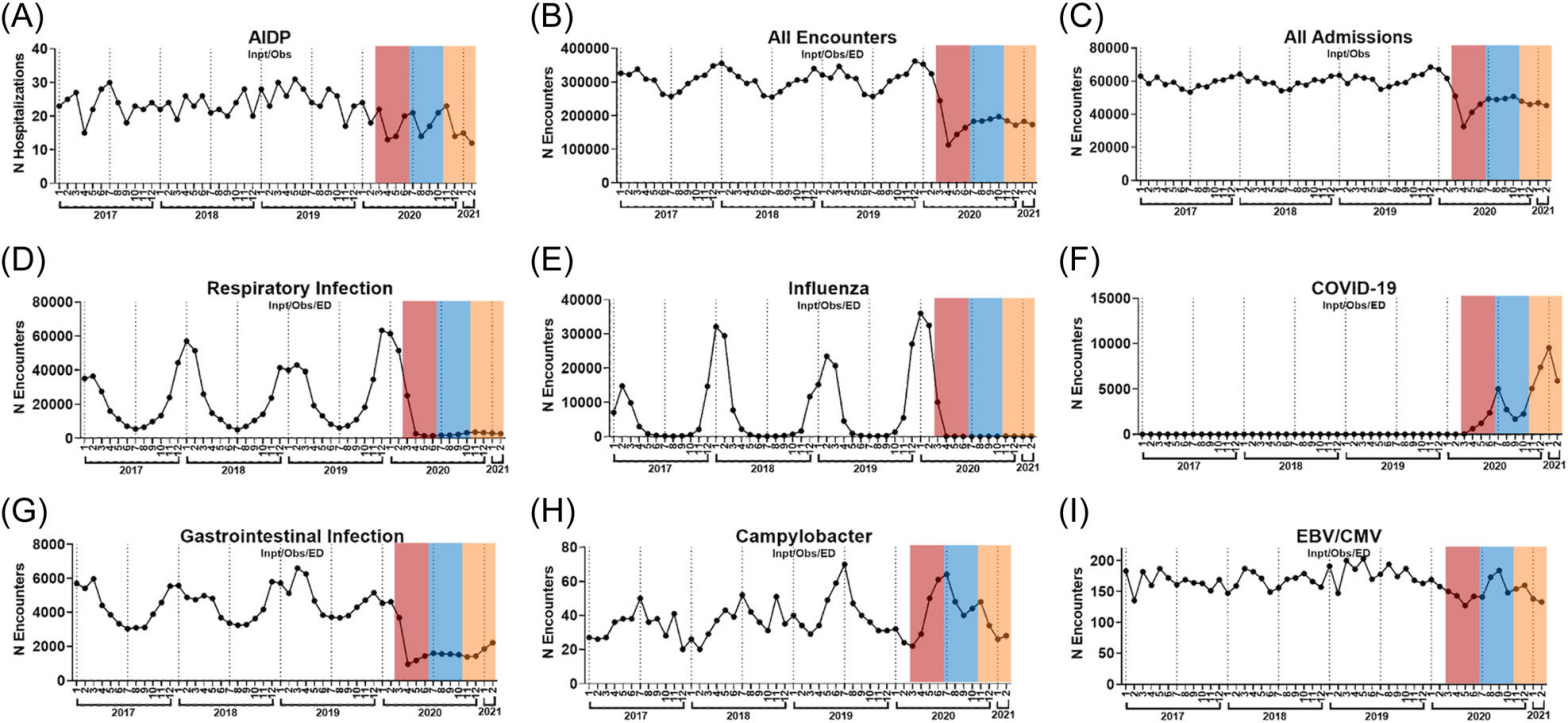
Encounters visualized over time from January 2017 to February 2021. Encounter types measured for each diagnosis are labeled with each graph. Shaded bars represent the four‐month epochs analyzed during the coronavirus disease 2019 (COVID‐19) pandemic. (A) Stable incidence of acute inflammatory demyelinating polyneuropathy (AIDP) encounters from 2017 to 2021 ranging between ~10 and 30/month. (B) All hospital encounters with seasonal winter trends until the beginning of the COVID‐19 pandemic. (C) Stable incidence of inpatient and observation encounters until the beginning of the COVID‐19 pandemic. (D, E, and G) Respiratory, influenza, and gastrointestinal infections with seasonal winter peaks until the COVID‐19 pandemic. (F) COVID‐19 infections/multisystem inflammatory syndrome in children (MIS‐C) encounters starting in 2020 and peaking in the winter of 2020 and early 2021. (H) Incidence of *Campylobacter jejuni* encounters with seasonal summer peaks. (I) Stable incidence of Epstein–Barr virus/cytomegalovirus (EBV/CMV) infections without the significant impact of the pandemic.

Pre‐COVID‐19, the incidence of AIDP encounters was not associated with the incidence of respiratory infections (*p* = 0.607), influenza (*p* = 0.954), gastrointestinal infections (*p* = 0.476), or *C. jejuni* (*p* = 0.186) (Figure 1). There was a weak association between AIDP and encounters for EBV/CMV (*r* = 0.34, *p* = 0.044).

### AIDP hospitalizations and association with COVID‐19 infections

The volume of COVID‐19 encounters was not associated with the volume of AIDP. Due to regional differences in COVID‐19 incidence, monthly incidence of COVID‐19/MIS‐C and AIDP were compared at individual institutions. No hospital had a positive correlation and 10% (4/39) had a statistically significant negative correlation (*p* < 0.05).

### Impact of COVID‐19 and social distancing/infection control on AIDP

We hypothesized that the impact of COVID‐19 on the spread of commonly acquired community infections was dramatic enough that if these were the main drivers of AIDP, there would be a proportional reduction in AIDP hospitalizations in a time‐dependent manner. Overall, during the COVID‐19 period, AIDP hospitalizations had a nonstatistically significant decrease of 11.5%–39.3%. This was similar to the statistically significant decrease in all encounters (ER, inpatient, and observation) for any diagnosis from March 2020 to February 2021 compared to expected (vs. pre‐COVID‐19) by 28.9%–46.1% (*p* < 0.03), and all admissions decreased by 14.2%–25.0% (*p* < 0.001) from July–February 2021 (*p* < 0.001) (Table 2 and Figure 1).

In contrast, when looking at infectious‐related encounters from March 2020 to February 2021, respiratory, gastrointestinal, and influenza infections decreased significantly compared to expected (for the same time of year pre‐COVID‐19) by 59.6%–90.1%, 51.5%–68.9%, and 54.5%–97.9%, respectively (Table 2 and Figure 1). When all time frames were included, there was a moderate association between AIDP encounters and all hospitalizations (*r* = 0.56), gastrointestinal infections (*r* = 0.55), and EBV/CMV (*r* = 0.51) (*p* < 0.001).

We compared AIDP to predetermined “controls” to determine if our methodology could detect effects related to changes in infectious trends or those likely to be impacted by changes in behaviors related to seeking care or referrals to the ER/hospital (i.e., canceled elective admissions, reduced hospitalization for mild illness). We expected index encounters for brain tumors or diabetic ketoacidosis to be less impacted by community‐acquired infections and asthma exacerbations to be highly reduced by a reduction in community‐acquired infections that can trigger asthma exacerbations. Index encounters for brain tumors decreased during the first four months by 33.4% (*p* = 0.003) and then nonsignificantly by 9.4%–20% (*p* = 0.51; 0.21). Diabetic ketoacidosis encounters trended down (14.9%; *p* = 0.25) during the first 4 months of the pandemic and then returned to baseline. In contrast, asthma exacerbation encounters decreased by 57.5%–83.3% (*p* > 0.02) (Table 2).

## Discussion

This study used a novel framework to evaluate the association between AIDP and seasonal infections from 2017 to early 2021. Further, the decrease in community‐acquired infections during the COVID‐19 pandemic allowed for an opportunity to determine if there was a similarly timed decrease in AIDP hospitalizations. Our study was able to capture the expected pre‐COVID‐19 seasonality of common community‐acquired infections and found no association with AIDP in children. More dramatically, COVID‐19 led to a substantial reduction in the rate of encounters related to infections, often by ~60%–98%. However, the reduction in AIDP was much more modest (12%–39%), mirroring the overall reductions in all hospitalizations (14%–25%).

Previous studies have found that two‐thirds of adult patients with AIDP report symptoms of respiratory or gastrointestinal infections before the onset of AIDP.^8^ In children, a recent study analyzed biosamples of patients with AIDP for serological evidence of recent infections. Children were more likely to have a positive immunoglobulin M (IgM) for *Mycoplasma pneumoniae* serology than adults. However, all of the children who were positive for *M. pneumoniae* appeared to have at least one other positive infectious serology (CMV or *C. jejuni*).^9^ Additionally, the detection of IgM antibodies was used as a marker for recent infection for *M. pneumonia*. Postinfectious IgM antibodies have been found in some individuals for up to nine months.^10^ Interestingly, researchers found that the rate of positive serology in the cohort was no different between those who did or did not report a recent illness. The association between AIDP and *C. jejuni* was further supported in this study; however, the link in children for other infectious causes was not as strongly demonstrated. In our study, we did not find a significant correlation between the incidence of respiratory or gastrointestinal community‐acquired infections and the incidence of AIDP prior to the COVID‐19 pandemic.

If common community‐acquired infections are not the triggers for AIDP, what are alternative triggers or etiologies? One possibility is that less common infections (like *C. jejuni*) cumulatively are the drivers of AIDP. However, these rare infections (if community acquired) would likely have been impacted by the same social distancing and infection mitigation efforts resulting from the COVID‐19 pandemic as those we measured. An example that follows this pattern more clearly is acute flaccid myelitis (AFM). AFM is thought to be associated with recent infections, in particular, enterovirus (EV)‐D68 and EV‐A71, though many other common infections have been associated with AFM. EV‐D68 and EV‐A71 have had peaks every two years (2014, 2016, and 2018), which are time‐locked to peaks of AFM.^9^, ^10^, ^11^, ^12^ During 2020, when an anticipated peak in EV did not occur, the rate of AFM remained similar to a nonpeak year for EVs.^13^

Another possibility is that the lag time from infection to AIDP symptoms could be longer (years), similar to that recently reported for EBV and MS.^11^ A large study of adults in the United States military found that the median time from estimated seroconversion to MS presentation was 7.5 years (range: 2–15). Additionally, they did not find a similar association with CMV or a difference in antibodies to a screen of viral peptides from known human viruses. How EBV infection increases the risk of MS is not fully understood, though hypotheses include molecular mimicry, immortalization of autoreactive B cells, EBV‐infected cells invading the central nervous system, T‐cell dysregulation, EBV‐associated inflammation, and interactions with human leukocyte antigen alleles.^14^

A third alternative is that reservoirs of infections that may trigger AIDP are less impacted by social distancing and infection prevention measures. This could include infections that are transmitted through contact with previously infected family members who intermittently shed virus (e.g., EBV)^15^ or through foodborne illness (e.g., *C. jejuni*). The Centers for Disease Control and Prevention reported that infections from foodborne pathogens did decrease during 2020, though by an average of 26% compared to 2017–2019.^16^ Recent studies including one published from South Korea evaluating the association of seasonality, viral trends, COVID‐19, and AIDP in all ages found a similar modest decrease in AIDP and only limited associations with seasons or specific pathogens. The strongest associations were with gastrointestinal pathogens that were less impacted by COVID‐19.^17^, ^18^

The reduction in AIDP during the pandemic era resembled that seen in overall hospitalizations, index brain tumor encounters, and diabetic ketoacidosis (not likely to be highly associated with infections). We found a substantial reduction in asthma exacerbations (58%–83%), which are triggered by respiratory viral infections in approximately 80% of cases,^19^ demonstrating that this methodology could identify a reduction in encounters for a diagnosis temporally associated with infectious trends without linking to infectious testing in each specific patient. Studies looking specifically at pediatric asthma exacerbations during COVID‐19 found that medication compliance decreased during this same time, which would have been expected to worsen exacerbations.^20^, ^21^

Our results are also consistent with a recent study finding that hospital encounters for children with migraine were decreased much more than encounters for status epilepticus or stroke.^12^ Combined, these data suggest that much of the AIDP reduction could be accounted for by decisions to not admit minor cases of AIDP, parental avoidance of ER evaluations for minor symptoms, and public health messaging to avoid unnecessary utilization of ER services. If true, the nonsignificant decrease in AIDP encounters during the pandemic likely underestimated the incidence of AIDP, as severe cases would likely have still been seen and hospitalized. Two studies—one in the United States by the Food and Drug Administration and one in Ontario, Canada—analyzed administrative databases to understand the background rates of “special adverse events of interest” in relation to vaccine safety. AIDP was reported in both studies to generally have had no or modest decreases similar to our study. In the United States, AIDP decreased slightly between 2019 and 2020 for adults but not significantly for children.^22^ In Ontario, comparing 2015–2019 to 2020, AIDP decreased by ~47% in children from 0 to 19 years of age.^23^

When specifically looking at COVID‐19/MIS‐C encounters, AIDP encounters nationally and at a single‐hospital level were either not associated or negatively associated with COVID‐19. We evaluated whether the increasing incidence of COVID‐19 infection (if it were a primary driver of AIDP) could have counterbalanced a decrease in the incidence of AIDP, such that the increase in COVID‐19 combined with the decrease in other infections could lead to an intermediate change in AIDP. However, during the first four months of the pandemic, we found that both COVID‐19 infections and other community‐acquired infections were low in children. The reduction is likely due to both reduced circulation of respiratory and gastrointestinal infections and avoidance of seeking care.^24^, ^25^ The incidence of AIDP was not significantly different during this time compared to later in the pandemic as COVID‐19 incidence increased. Our findings are consistent with an epidemiological and cohort study in the United Kingdom that did not find an association between COVID‐19 infection and AIDP,^19^ but they conflict with reports linking COVID‐19 and AIDP.^5^, ^6^, ^7^

We found a weak positive correlation between AIDP and EBV/CMV (before and during the COVID‐19 era) and a moderate correlation for gastrointestinal infections (only during the COVID‐19 era), which requires further investigation. It is likely that the incidence of hospital encounters for EBV/CMV underestimates the local incidence as many infections are mild and do not require testing or hospitalization (unless patients are immunocompromised). Thus, this may not represent community spread. Alternatively, influenza, respiratory syncytial virus, and COVID‐19 are more frequently tested for and assigned a diagnosis. The weak association with gastrointestinal infections only after including the COVID‐19 era is likely driven by an overall trend slightly downward since the beginning of the study time for all admissions, gastrointestinal infections, and EBV/CMV rather than a time‐dependent association. Importantly, AIDP did not show similar seasonal fluctuation as gastrointestinal illness encounters, nor a similar ~50%–70% decrease during the COVID‐19 pandemic.

### Limitations

Our study has several limitations. The diagnoses studied were obtained from medical billing codes, with the assumption that community prevalence was proportionately represented by hospital encounters. Diagnosis and treatment of AIDP were determined by ICD‐10 coding and billing for medications/treatments. Importantly, the number of encounters for AIDP and commonly administered treatments were in the expected ranges. Plausible alternative diagnoses that could be confused for AIDP were excluded a priori and were a small percentage of encounters. Additionally, these inaccuracies would have been expected to be constant during the course of the study. We utilized hospital encounters as a proxy for infection incidence in the community. We likely underestimated the community spread of infections that are rare or unlikely to be tested. This analysis would not easily identify local small outbreaks that could correlate with a few cases of AIDP. Additionally, it is possible that a lower proportion of all community‐acquired infections are treated at academic centers, as compared to the proportion of AIDP cases, which are more commonly treated at larger academic centers and therefore relatively over‐represented in the PHIS database.

## Conclusions

In summary, we found no strong association between community‐acquired infections and the incidence of AIDP in children at the national level, nor locally for COVID‐19/MIS‐C. These results raise questions about a causal link between a recent antecedent community‐acquired infection and AIDP. Alternative causes for the onset of AIDP outside of infections should be considered, including a genetic predisposition for the development of autoantibodies such as is found in type 1 diabetes.^20^ Additionally, this study's methodology may allow for similar investigations of other conditions that are hypothesized to be post‐ or peri‐infectious. Future studies using infectious trends for specific infections and surveillance for neurological (or other disorders) may improve spatial and temporal resolution to identify causes of peri‐infectious conditions.

## Author Contributions

**Hannah Gilbert**: Conceptualization; methodology; project administration; writing—original draft; writing—review and editing. **Nicholas S. Abend**: Conceptualization; methodology; writing—review and editing. **Melissa Hutchinson**: Conceptualization; writing—review and editing. **Ricka Messer**: Conceptualization; writing—review and editing. **Mahendranath Moharir**: Conceptualization; writing—review and editing. **Kendall Nash**: Conceptualization; methodology; writing—review and editing. **Jamie Palaganas**: Conceptualization; writing—review and editing. **Juan Piantino**: Conceptualization; writing—review and editing. **Samir S. Shah**: Conceptualization; methodology; writing—review and editing. **Matt Hall**: Data curation; formal analysis; methodology; resources; visualization; writing—original draft; writing—review and editing. **Elizabeth Wells**: Conceptualization; methodology; writing—review and editing. **Craig A. Press**: Conceptualization; formal analysis; methodology; project administration; supervision; writing—original draft.

## Pediatric Neurohospitalist Work Group

Nicholas S. Abend (MD), Melissa Hutchinson (MD), Ricka Messer (MD, PhD), Mahendranath Moharir (MD), Kendall Nash (MD), Jamie Palaganas (MD), Juan Piantino (MD), Elizabeth Wells (MD), Craig A. Press (MD, PhD).

## Conflict of Interest

The authors declare no conflicts of interest.

## Supporting information

Supporting Information.

## Data Availability

The authors state that anonymized data on which the article is based will be shared by request from any qualified investigator.
